# Multitarget Molecular Imaging in Metastatic Castration Resistant Adenocarcinoma Prostate Cancer with Therapy Induced Neuroendocrine Differentiation

**DOI:** 10.3390/diagnostics12061387

**Published:** 2022-06-03

**Authors:** Joel Vargas Ahumada, Sofía D. González Rueda, Fabio A. Sinisterra Solís, Quetzali Pitalúa Cortés, Liliana P. Torres Agredo, Jimenez Ríos Miguel, Anna Scavuzzo, Irma Soldevilla-Gallardo, Miguel A. Álvarez Avitia, Nora Sobrevilla, Francisco Osvaldo García Pérez

**Affiliations:** 1 Nuclear Medicine and Molecular Imaging Department, National Cancer Institute, Tlalpan, Mexico City 14080, Mexico; drjoelvargas7@gmail.com (J.V.A.); denissegr205@gmail.com (S.D.G.R.); fasisoob@hotmail.com (F.A.S.S.); quecho70@hotmail.com (Q.P.C.); irmasoldevilla81@hotmail.com (I.S.-G.); 2 Nuclear Medicine Department, Universidad Autónoma de Bucaramanga, Bucaramanga 680002, Colombia; lilipa.torres@gmail.com; 3 Urological Oncology Department, National Cancer Institute, Tlalpan, Mexico City 14080, Mexico; incanurologia@gmail.com (J.R.M.); annasc80@gmail.com (A.S.); 4 Medical Oncology Department, National Cancer Institute, Tlalpan, Mexico City 14080, Mexico; migal720613@yahoo.com.mx (M.A.Á.A.); nsobrevilla@gmail.com (N.S.)

**Keywords:** prostate cancer, neuroendocrine differentiation, PET/CT, molecular imaging

## Abstract

Neuroendocrine differentiation of prostate cancer (NEDPC) includes de novo presentation and secondary to epigenetic changes, referred as therapy-induced neuroendocrine prostate cancer (t-NEPC). Molecular imaging with prostate-specific membrane antigen (PSMA) and somatostatin analogues positron emission tomography (PET/CT) in NEDPC have not been validated. ^18^F-FDG (fluorodeoxyglucose) PET/CT has numerous limitations in prostate cancer (PCa) and the utility in NEDPC has only been reported in a few series of cases. The objective of this study is to compare the lesions detection rate of the three radiotracers in metastatic t-NEPC patients. (1) Material and Methods: Retrospective evaluation of patients with prostate adenocarcinoma treated with androgen deprivation therapy, chemotherapy, a novel androgen receptor pathway inhibitor or a combination of them and a second tumour biopsy confirming t-NEPC was made. All patients underwent ^18^F PSMA-1007, ^18^F AlF-NOTA-Octreotide, and ^18^F-FDG PET/CT. Evaluation of positive lesions was determined and SUVmax of each radiotracer was estimated and correlated with computer tomography (CT) findings. (2) Results: A total of eight patients were included. The mean time from diagnosis of prostate adenocarcinoma to t-NEPC was 28.2 months, with a mean serum specific prostate antigen (PSA) of 16.6 ng/dl at the time of NEPC diagnosis. All patients were treated with antiandrogen therapy and 87.5% with chemotherapy. A total of 273 lesions were identified by CT from which 182 were detected by ^18^F-FDG PET/CT, 174 lesions by ^18^F PSMA-1007, and 59 by ^18^F AlF-NOTA-Octreotide. An interpatient analysis of the lesions was performed and dual tracer ^18^F-FDG PET/CT and ^18^F PSMA-1007 PET/CT detected a total of 270/273 lesions (98.9%). (3) Conclusions: NEDPC patients demonstrated wide inter and intrapatient molecular imaging heterogeneity within the three radiotracers. ^18^F-FDG detected most lesions in t-NEPC among all radiotracers, especially in visceral sites; ^18^F PSMA-1007 detected more bone lesions. ^18^F AlF-NOTA-Octreotide showed no significant utility.

## 1. Introduction

Prostate cancer (PCa), a prevalent neoplasia in men, usually exhibits slow growth and progression behaviour and it is one of the deadliest neoplasms in the world. The prevalence and incidence of this neoplasm have increased, making it a health problem worldwide [1,2].

The prostate gland is composed of stromal cells, stem cells and epithelial cells, which are predominantly luminal, basal, and, to a lesser degree, neuroendocrine cells. In most cases, prostate cancer originates in the luminal cells, although, due to intratumoural heterogeneity, other cell subgroups can coexist according to their molecular profile, which directly impacts the prognosis [3,4]. Management of PCa has evolved over the years as we escalate the level of comprehension of the disease. Typical systemic treatments focus on targeting androgen receptor (AR) signalling by inhibiting androgen production (surgical or medical castration) and/or blocking AR function with competitive inhibitors. However, the therapeutic efficacy is temporary, and patients will inevitably experience disease recurrence and progress to castration-resistant prostate cancer (CRPC). It is well established that androgen receptors are essential for PCa development, in such a way that therapeutic strategies have sought to inhibit androgenic synthesis at an intratumourally level (i.e., abiraterone) or in a more efficient way, inhibit AR signalling (i.e., enzalutamide) [5].

As time has passed and these types of therapies have been used more frequently, an intriguing phenomenon has been described with the clinical observation of how advanced PCa evades androgen inhibitors and other therapies. It has been validated that PCa tumours respond to antiandrogen therapy (ADT) and, afterwards, some cells thrive into focal neuroendocrine differentiation of CRPC, this phenomenon, also known as t-NEPC, has generated great interest among the scientific community, especially because of the mechanisms that are activated by neoplastic cells: the epithelial-mesenchymal transition would be one of the main mechanisms suspected in these processes [6,7].

Neuroendocrine prostate carcinoma (NEPC) is an aggressive subtype of prostate cancer characterized by a lack of responsiveness to hormonal therapies and an overall poor prognosis that can arise de novo, which is rare and ranges from 0.5–2% of total PCa cases, but much more commonly arises after hormonal therapy for prostate adenocarcinoma, reported at a much higher rate, ranging from 17–30% [8]. The genotypic characteristics of this prostate cancer variant are highly variable and also show the expression of genes that are used as markers for neuroendocrine (NE) differentiation, e.g., synaptophysin (SYP), chromogranin A (CgA), and enolase 2 (ENO2) [9].

The biological characteristics of this type of cancer present a diagnostic challenge for conventional imaging modalities, such as computer tomography (CT) or magnetic resonance imaging (MRI), especially when patients have been exposed to therapies in which it is impossible to discriminate active disease from anatomical alterations conditioned by therapy (i.e. sclerotic bone lesions) [10]. Molecular imaging with ^68^Ga/^18^F-PSMA PET/CT is the upcoming imaging modality for staging, restaging and response assessment of prostate cancer [11]. This new generation of imaging has shown better diagnostic accuracy when compared with conventional imaging, such as CT or bone scan, particularly in high-risk PCa patients [12]. However, the expression of PSMA may be epigenetically downregulated and this phenomenon leads to false-negative results in patients with these neuroendocrine characteristics.

It is documented that well-differentiated neuroendocrine tumour cells express somatostatin receptors (SSTR), consequently, they can be detected by PET tracers for SSTR like ^68^Ga-DOTATOC, but their application in prostate cancer is not well understood [13]. The use of fluorodeoxyglucose (FDG) PET/CT in PCa is limited due to the metabolic behaviour of this entity, characterized by low glucose consumption and the use of other metabolic pathways such as fatty acids and fructose [14,15]. However, Spratt et al. demonstrated that ^18^F–FDG PET/CT has clinical utility in the metastatic evaluation of NEPC due to the phenotypic characteristics, which have a much higher glucose consumption being a high-grade neuroendocrine subtype [16]. Given this evidence, it is important to identify the best imaging biomarker that can be used in suspected t-NEPC, especially because they may not present serum prostate-specific antigen (PSA) elevation, and, if any, it ought to be subtle. In addition, it is of great importance to initiate an appropriate therapeutic strategy due to the rapid progression and poor overall survival linked with these histologies [17]. As the clinical utility of PSMA, somatostatin analogues and FDG PET/CT has not been yet well comprehended, we performed a comparison of each one of them in histologically proven NEDPC patients.

## 2. Materials and Methods

### 2.1. Study Population

This is a retrospective study of eight patients with NEDPC who were referred to the National Cancer Institute (INCan) between 2018 and 2021. All patients underwent ^18^F PSMA–1007, ^18^F AlF-NOTA-Octreotide and ^18^F-FDG PET/CT within 5 weeks, with the following inclusion criteria:

(a) Patients with metastatic CRPC (CRPCm) and histologically proven neuroendocrine prostate cancer.
(b) First initial diagnosis biopsy-proven with adenocarcinoma PCa with a second biopsy that confirmed NE differentiation with immunohistochemical markers (chromogranin and synaptophysin).
(c) Prior treatment based on androgen deprivation therapy, chemotherapy, novel androgen receptor pathway inhibitor or a combination of each one of them.

Exclusion criteria:

(a) Patients who had other primary malignancies at the time of examination.
(b) Non-histopathological NEPC confirmation. NEPC was defined by the presence of either pure small/large-cell carcinoma (by tumour morphology) or mixed histology with both adenocarcinoma and small/large-cell neuroendocrine carcinoma along with positive neuroendocrine immunohistochemical markers [17].

### 2.2. Image Acquisition

PET/CT Whole-body was acquired from top of head to mid-thigh approximately 60 min after the intravenous injection of each radiotracer; ^18^F PSMA–1007 (370 MBq), ^18^F AlF-NOTA-OCTREOTIDE (259 MBq) and ^18^F-FDG (370 MBq) within 5 weeks, according to the clinical standard protocol for tumour imaging. A Biograph mCT 20 Excel PET/ CT scanner was used (Siemens Healthineers, Knoxville, TN, USA). The PET reconstruction datasets were 400 × 400 matrix (pixel size: 1.5625 × 1.5625 × 2.78 mm^3^) with Time of Flight (TOF) OSEM algorithms with 21 subsets and 3 iterations, followed by a 6-mm Gaussian filter. CT was acquired using 140 mA, 130 kV, 5 mm width, and a 1 mm pitch.

### 2.3. Image Analysis

Images from the three radiotracers were simultaneously evaluated by two experienced nuclear physicians to define the positivity of the lesions. Lesions were classified by regions in the prostate, seminal vesicles, lymph nodes, bone, and visceral metastases (lung, liver, and mesenteric). The criterion of positivity was focal uptake areas higher than in the surrounding tissue background. The standardized value of maximum uptake (SUVmax) of lesions was measured with a region of interest (ROI) using a syngo.via workstation (Siemens Healthineers).

### 2.4. Statistical Analysis

Results are shown as mean ± SD or as frequencies (%). For comparison of continuous variables, the two-tailed Student’s T-test was obtained for unpaired data. The x2 test was applied to compare nominal variables. Kaplan–Meier graphs were made. All statistical analyses were performed using STATA 14, with a *p* value < 0.05 considered statistically significant.

## 3. Results

**Figure 1:**
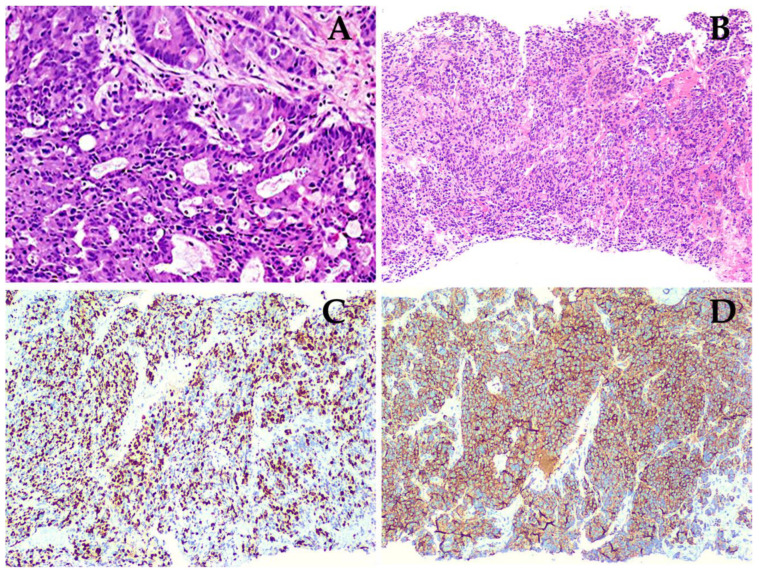
Patient number 2: (**A**) Image of a slide of the initial biopsy, with a 20x zoom, stained with hematoxylin and eosine (H&E), revealing a prostate mucinous adenocarcinoma, in which mucinous component is predominantly composed of cribriform glands, with a Gleason score of 8 (4 + 4). (**B**) Image of a pulmonary metastasis biopsy of the same patient, performed after therapy, also stained with H&E, with a 10x zoom lens, showing a neoplastic lesion, with an organoid pattern (sheet–like tumor nests); cells are large, with a variable shaped nuclei, fine granular chromatin, and a small nucleolus, scant clear to eosinophilic cytoplasm, some cells with marked pleomorphism, atypical mytotic figures, and apoptotic bodies. (**C**) Ki67 immunohistochemistry (IHC), evidentiating a positive nuclear reaction in 70% of the cells, with an intense and diffuse granular cytoplasmatic staining. (**D**) Synaptophysin positive IHC.

Eight patients who met the criteria of interest were selected, the mean age at diagnosis was 71 years with an interquartile range (IQR) of 62.5–81.5 years, and mean PSA of 16.6 ng/dl (IQR 2.75–138.65) at the time of t-NEPC diagnosis. According to histology, 87.5% (*n* = 7) were prostate adenocarcinoma and 12.5% (*n* = 1) presented with mucinous prostate adenocarcinoma (Figure 1). In the Gleason scoring system, 50% (*n* = 4) of the patients presented a Gleason 9 (4 + 5) and an ISUP grade greater than 9 in 75% (*n* = 6). When evaluating the initial treatment of the 8 patients, only 37. 5% (*n* = 3) underwent surgical treatment, which, in 12.5% of the cases, was radical prostatectomy and 25% underwent transurethral resection of the prostate (TURP); 100% of patients received hormonal treatment and 87. 5% (*n* = 7) received chemotherapy. When performing a second biopsy, 75% (*n* = 6) presented neuroendocrine carcinoma, and 25% large cell neuroendocrine carcinoma (see Table 1). The eight biopsy cases were complemented with immunohistochemical studies, of which 100% were positive for synaptophysin and chromogranin, other markers were performed in 75% of patients (see Table S1 Supplementary Materials).

Mean time for neuroendocrine differentiation was 28.28 months (95% CI 12.8–42.75). The mortality incidence rate was 37.5% (*n* = 3). Mean overall survival (OS) was 13.25 months (95% CI 4.84–21.65). The Kaplan–Meier survival analyses for the patients are found in Figures S1 and S2 Supplementary Materials.

The ^18^F–PSMA-1007, ^18^F–FDG and ^18^F AlF-NOTA-Octreotide PET/CT were performed within 5 weeks. A total of 273 lesions were identified. ^18^F–FDG was positive in 182 lesions, ^18^F–PSMA-1007 positive lesions were 174, and 59 ^18^F AlF-NOTA-Octreotide positive lesions were identified. The most representative lesions were found at bone level in 60.8% (*n* = 166), visceral in 30.04% (*n* = 82), followed by lesions in lymph nodes (6.59%, *n* = 18), prostate bed/prostate and seminal vesicles in 1.83% (*n* = 5) and 0.73% (*n* = 2), respectively. In visceral sites, the organs involved were liver in 46.34% (*n* = 38), lungs in 52.44% (*n* = 43) and mesentery in 1.2% (*n* = 1). The most frequent sites in which lymph nodes were involved were pelvis (50%), retroperitoneum (44.4%), and inguinal (5.56%).

**Figure 2:**
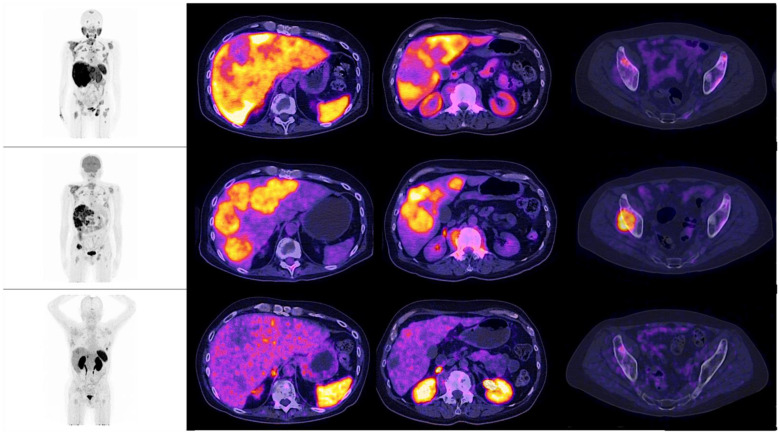
Patient number 4, a 54-year-old male, with an initial histopathological report of prostate adenocarcinoma, and a Gleason score of 9 (4 + 5). He was treated with surgical transurethral resection of the prostate + androgen deprivation therapy + chemotherapy. Site of second biopsy was post-surgical prostate bed, with a confirmatory report of neuroendocrine carcinoma (positive immunohistochemistry for chromogranin and synaptophysin). Time lapse to neuroendocrine differentiation was 15 months, with a PSA value of 137.3 ng/dL when NED was detected. In the illustrations, multiple hepatic lesions are appreciated, all of them with an increase of the glycolytic metabolism, with no evidence whatsoever, of expression of PSMA nor somatostatin receptors, by molecular imaging.

**Figure 3:**
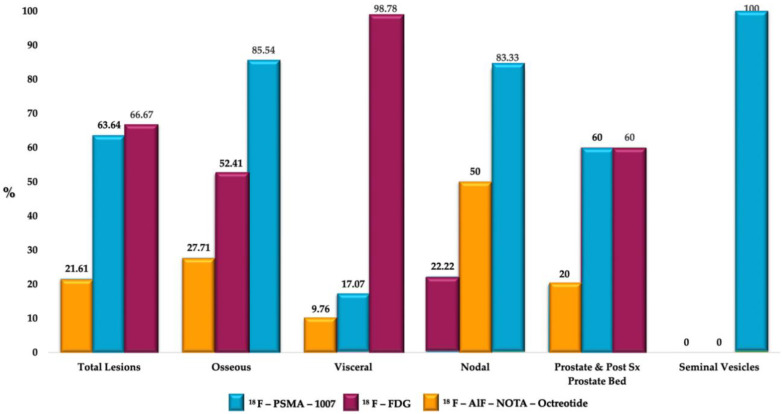
Percentage of lesions detected by ^18^F-PSMA-1007, ^18^F-FDG and ^18^F-AlF-Nota Octreotide by regions.

When observing the lesions and the percentage of uptake by a semi-quantitative assessment, which was determined by SUVmax for each radiopharmaceutical, it was found that the positive uptake of ^18^F–PSMA-1007 is greater at bone lesions, lymph node and seminal vesicles compared with the other radiotracers. Conspicuously, FDG was identified as the best radiotracer for the overall identification of lesions, with an outstanding detection of visceral metastases (see Figure 2 and Figure 3).

**Figure 4:**
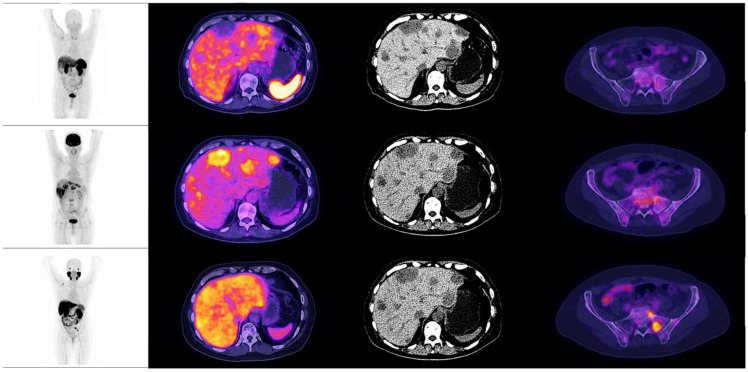
Patient number 7, a 67-year-old male, with an initial histopathological report of prostate adenocarcinoma, Gleason score 8 (4 + 4). Treated with androgen deprivation therapy + chemotherapy. Site of second biopsy was in a liver lesion, with a confirmatory histopathological report of neuroendocrine carcinoma (positive immunohistochemistry for chromogranin and synaptophysin). Time lapse to neuroendocrine differentiation (NED) was 48 months, with a PSA value of 19.8 ng/dL at the time of diagnosis. The image shows the maximum intensity projection of the three radiotracers (^18^F AlF-NOTA-Octreotide, ^18^F–FDG and ^18^F–PSMA-1007) with multiple hepatic lesions and focal uptake of ^18^F-FDG PET/CT; PSMA and somatostatin analogues didn’t evidentiate radiotracer uptake. The image also displays bone metastases in left iliac bone and sacrum, both PSMA avid.

**Figure 5:**
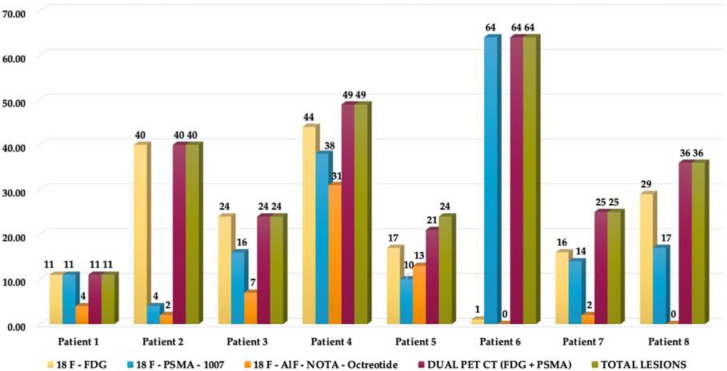
Lesions detection analysis per patient. Total lesions detected by CT were 273 (100%). Total lesions detected by dual PET/CT (^18^F-FDG + ^18^F-PSMA-1007) were 270 (98.9%).

**Figure 6:**
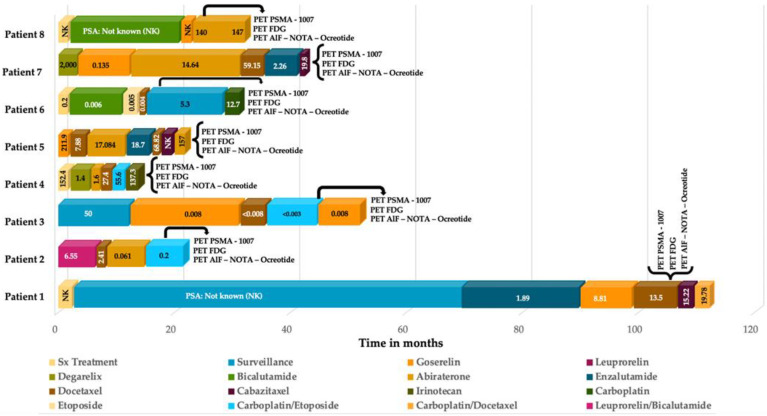
Therapy and PSA correlation analysis per patient.

**Figure 7:**
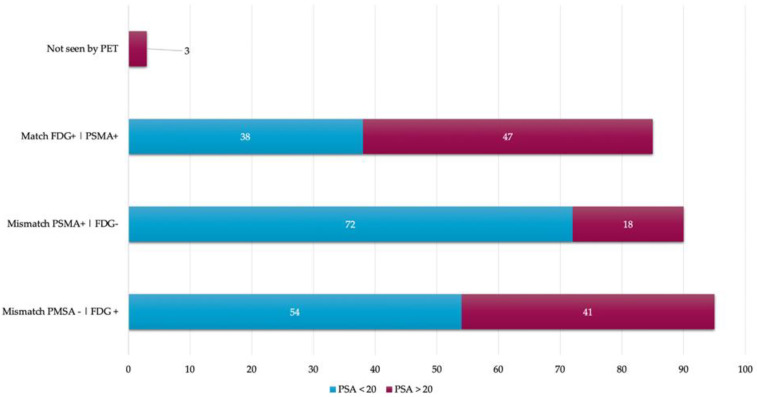
Lesions detection rate according to PSA levels.

An interpatient analysis of the lesions was carried out: it demonstrated that out of the 273 lesions, 185 were discordant, and 85 concordant between ^18^F–PSMA-1007 and ^18^F-FDG PET/CT. When making a summation of the discordant and concordant lesions between both radiotracers, results demonstrate that dual ^18^F–PSMA-1007 and ^18^F-FDG PET/CT detected a total of 270/273 which corresponds to 98.9% of all lesions visualized by CT. ^18^F AlF-NOTA-Octreotide PET/CT showed a low detection rate (See Figure 4 and Figure 5). A therapy and PSA correlation analysis per patient was also performed, as well as lesion detection rate according to PSA levels (Figure 6 and Figure 7).

The 273 lesions were observed by CT, however, only 22.34% (*n* = 61) of them were measurable according to RECIST 1.1 criteria. The mean size was 18 mm (IQR 14–37 mm).

**Figure 8:**
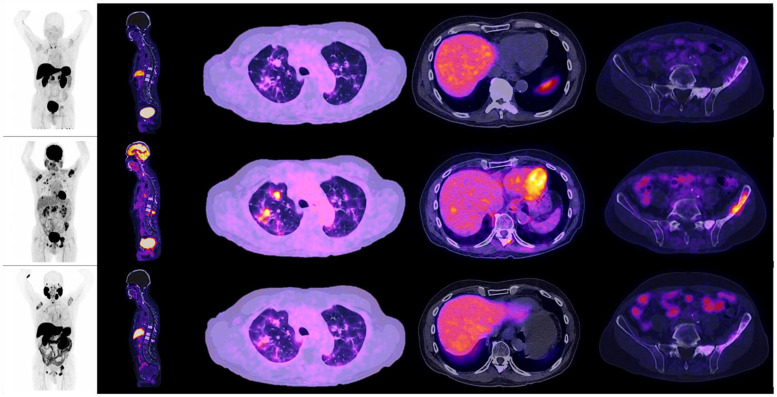
Patient number 3, a 77-year-old male, with an initial histopathological report of prostate adenocarcinoma, Gleason score 9 (4 + 5). Treated with androgen deprivation therapy + chemotherapy. Site of second biopsy was in left iliac bone, with a confirmatory histopathological report of neuroendocrine carcinoma (positive immunohistochemistry for chromogranin and synaptophysin). Time lapse to neuroendocrine differentiation was 48 months, with a PSA value of 0.003 ng/dL at the time of diagnosis. The image shows the maximum intensity projection of the three radiotracers (^18^F AlF-NOTA-Octreotide, ^18^F–FDG and ^18^F–PSMA-1007) with multiple lung nodules, a single liver metastasis and osteoblastic lesions with high glycolytic metabolism in molecular imaging. ^18^F–PSMA-1007 and ^18^F AlF-NOTA-Octreotide PET/CT showed diffuse uptake of the radiotracer in described lesions.

**Figure 9:**
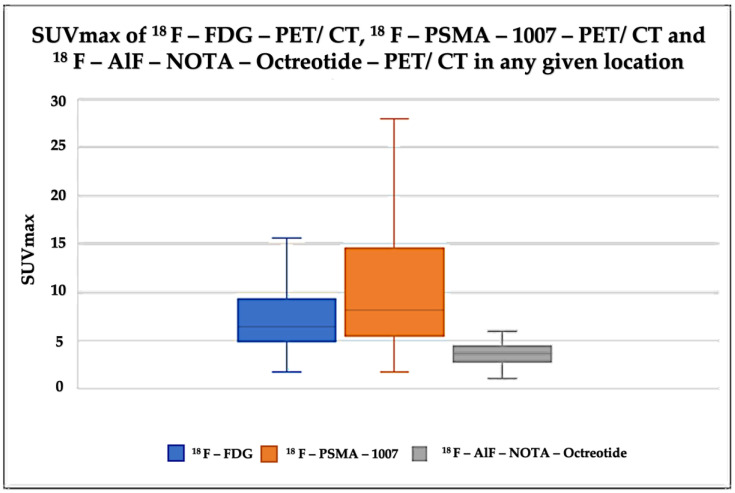
Box-plot diagram of SUVmax in the different radiopharmaceuticals.

The analysis revealed that the mean SUVmax for ^18^F-FDG was 6.4 (IQR 4.9–9.3), 6.75 for ^18^F–PSMA-1007 (IQR 4.5–9.4) and 4.6 for ^18^F AlF-NOTA-Octreotide (IQR 2.8–4.4) (see Figure 8 and Figure 9).

## 4. Discussion

NEPC is an aggressive subtype of prostate cancer that may arise de novo or manifest in the later stages of prostate cancer, characterized by focal neuroendocrine features due to epigenetic alterations conditioned by multiple therapies. This subgroup of patients is often treated with chemotherapy (platinum-based), given their pathological and molecular similarities with small cell lung carcinoma (SCLC). Under this premise, the National Comprehensive Cancer Network (NCCN) guidelines currently take into consideration a new biopsy of metastatic lesions, in any patient with CRPC to look for t-NEPC transformation [18]. Rapid worsening of performance status, low serum PSA despite high tumour burden, visceral metastases (particularly lung and liver) and osteolytic bone metastases, should lead physicians to suspect NE differentiation of prostate cancer either de novo or as CRPC [19].

Molecular imaging characteristics in t-NEPC are poorly defined; however, they may help guide further diagnosis, knowing when to perform a biopsy, therapy of choice, and assessment to therapeutic response. In this study, we reviewed the clinical and molecular imaging features of a cohort of patients with t-NEPC, to provide insights into lesions detection, cellular heterogeneity, prognosis, and other variables that may aid in clinical management.

Multitarget molecular imaging is now available for the evaluation of prostate cancer and neuroendocrine differentiation. PSMA-targeted inhibitors imaging and therapy are transforming the landscape of PCa management [20,21]. Despite the great impact and evolution that PSMA has had, there are clinical reports that suggest that PSMA-targeted imaging does not visualize NEPC tumours. Studies have demonstrated that AR regulate the expression of FOLH1, which is the gene responsible for encoding the transmembrane protein prostate-specific membrane antigen; also, the induction of lineage plasticity by AR inhibition leads to neuroendocrine differentiation and PSMA suppression [22,23]. In our study ^18^F–PSMA–1007 PET/ CT detected a significant number of lesions, especially bone metastases, with an even better detection rate than ^18^F–FDG PET/ CT at these specific sites, which could be possibly explained by the identification of mixed histologic subtypes. Tumoural heterogeneity plays an important role in this context. Although patients in this study underwent biopsy of a single metastatic site, and heterogeneity across different metastatic sites in the same patient was not evaluated by biopsy, molecular imaging findings suggest that mixed histologies were prevalent in most of our patients and that an adenocarcinoma component could lead to higher affinity to osseous sites. Rahul Aggarwal et al., in a multi-institutional prospective study, demonstrated that treatment-induced small-cell neuroendocrine prostate cancer is present in nearly one-fifth of patients with mCRPC (which is associated with shortened survival), that neuroendocrine differentiation is a heterogeneous process, and that the identification of molecular characteristics may provide a basis for tumour classification, clinical recommendations and future development of therapies [24].

Assessing tumoural heterogeneity by molecular imaging using ^18^F –FDG PET/ CT, has been studied in some recent case reports, with excellent metastases detection rates (up to 95%), specifically the visceral and nodal metastases in NEPCs [25,26]. Interestingly, Thang et al. screened patients with ^68^Ga-PSMA-11 and ^18^F-FDG PET, identifying subjects with low PSMA expression and high glycolytic metabolism. These same findings were correlated in a different study, with a serum level of PSA, where it was exhibited that in men with low PSA levels, ^18^F-FDG uptake was higher [27,28]. In our population, ^18^F-FDG PET/CT detected the most lesions among all radiotracers, especially in visceral sites with a 98% detection rate, in which ^18^F–PSMA–1007 PET/ CT only detected 17%. This correlates with findings presented by Bakht et al., where they evaluated the differential expression of glucose transporters and hexokinases in prostate cancer with a neuroendocrine gene signature and demonstrated that neuroendocrine gene signature associates with a distinct transcriptional profile of GLUTs and hexokinases. PSMA suppression correlates with GLUT12 suppression and glucokinase upregulation. Alteration of genes that are associated with a higher ^18^F-FDG uptake, correlated positively with higher glucose uptake in both AR and PSMA suppressed tumours [29]. These results show that aggressive prostate cancer subtypes transform into higher glucose metabolism tumours and make it possible for ^18^F-FDG PET/CT to detect more lesions than PSMA PET/ CT.

Another novel radiotracer that has gained usage is positron-emitting somatostatin analogue, which bind to somatostatin receptors (SSRTs) with high affinity to well-differentiated neuroendocrine tumours [30]. Usmani et al. reported a case that showed the significance of somatostatin receptors scintigraphy in the detection of neuroendocrine differentiation of metastatic prostate cancer [31]. In another study DOTATATE PET/ CT showed high uptake in mCRPC and in suspected or known NEPC patients; also, Gofrit et al., studied a metastatic group of patients, previously treated with ADT, where 50% of patients showed a moderate or high tracer uptake with ^68^Ga-DOTATATE PET/ CT [13,32]. These findings do not correlate with our cohort of patients. Our results showed that ^18^F AlF-NOTA-Octreotide PET/CT in t-NEPC patients had low detection rate, as well as low uptake of the radiotracer in metastatic lesions, suggesting somatostatin analogues PET/ CT has no significant utility for diagnostic nor benefit from radionuclide therapy in this group of patients. Radionuclide therapy possibility has also been studied by Amir Iravani et al. in five patients with t-NEPC in which none of them were suitable for PSMA or SSTR-based radionuclide therapy, neither a combination of them, because none of the radiotracers managed to target all sites of the disease, due to the discordant ^18^F-FDG-avid disease sites and tumour heterogeneity [33].

In our results, the interpatient analysis of the lesions showed alluring data: there was a total of 185 discordant and 85 concordant lesions between ^18^F–PSMA-1007 and ^18^F-FDG PET/CT. When making a summation of the discordant and concordant events of both radiotracers, it was obtained that dual PET/CT tracer detected 98.9% of all the lesions visualized by CT; only three lesions were not visualized by PET, which corresponded with sclerotic bone areas that could be explained by non-active disease. It was also demonstrated that only 22.34% of the lesions observed by CT were measurable according to RECIST 1.1 criteria, which represents a problem when assessing treatment response in this group of patients; thus, molecular imaging may have a better performance in this setting.

We recognize several limitations of our study, including the retrospective non-randomized design, the limited amount of patients, few analysed metastatic biopsies with molecular data available and the lack of genomic profiles. Although we had a narrow number of patients evaluated, this was compensated by the quantity of lesions observed. This study describes, to the best of our knowledge, the experience with the biggest cohort regarding multitarget molecular imaging in t-NEPC patients, in order to provide new insights into the clinical hallmarks. Finally, based on these results, we consider that ^18^F-FDG PET/CT could be performed at the moment that t-NEPC is clinically suspected, especially in patients with visceral lesions with no PSMA expression, in order to evaluate tumoural heterogeneity, guided biopsy, assessment of treatment response, and overall management of patients with NEPC. It is also important to state that biopsy may lead to diverse complications, essentially in susceptible organs (lung, liver, etc.), not to mention its low sensitivity when it comes to bone lesions, along with the high costs implied; therefore, looking forward into the near future, molecular imaging with PET/ CT could manage to improve diagnostic accuracy, with the advantage of a non-invasive procedure, but future prospective studies should be performed.

## 5. Conclusions

T-NEPC patients demonstrated wide inter and intrapatient molecular imaging heterogeneity by ^18^F–PSMA-1007, ^18^F AlF-NOTA-Octreotide, and ^18^F-FDG PET/CT. ^18^F-FDG detected most lesions among all radiotracers, especially in visceral sites, even though ^18^F–PSMA-1007 detected more bone lesions. ^18^F AlF-NOTA-Octreotide showed no significant utility. This study shows that dual tracer PSMA and FDG PET/CT could be performed to identify tumoural heterogeneity and active disease in clinically t-NEPC suspected patients.

## Figures and Tables

**Table 1:** General characteristics of the patients.

Patient	Age (Years)	Initial HPE ^1^	Gleason	Treatment	Type of Treatment	Second HPE ^1^	Site of 2° Biopsy	Time to NED ^2^	PSA ^3^ (ng/dL)
1	75	Pac ^4^	9 (4 + 5)	Sx ^6^ RP ^7^ + ADT ^9^+ ChT ^10^	Enzalutamide, Gosereline, Docetaxel, Cabazitaxel, Carboplatin	NEC ^11^	Bone	108	13.5
2	59	PMAc ^5^	8 (4 + 4)	ADT + ChT	Bicalutamide, Leuprorelin, Docetaxel, Abiraterone, Etoposide, Carboplatin	LCNEC ^12^	Lung	14	0.2
3	77	PAc	9 (4 + 5)	ADT + ChT	Goserelin, Docetaxel, Etoposide, Carboplatin	NEC	Bone	48	0.003
4	54	PAc	9 (4 + 5)	Sx TURP ^8^ + ADT + ChT	Degarelix, Abiraterone, Docetaxel, Carboplatin, Etoposide, Irinotecan	NEC	Post Sx Bed/Prostate	15	137.3
5	66	PAc	9 (4 + 5)	ADT + ChT	Goserelin, Docetaxel, Abiraterone, Enzalutamide, Cabazitaxel	NEC	Prostate	24	157
6	86	PAc	9 (5 + 4)	Sx TURP + ADT + ChT	Leuprorelin, Bicalutamide, Etoposide, Docetaxel, Carboplatin	LCNEC	Post Sx Bed/Prostate	13	5.3
7	67	PAc	8 (4 + 4)	ADT + ChT	Degarelix, Goserelin, Abiraterone, Docetaxel, Enzalutamide, Cabazitaxel	NEC	Liver	48	19.8
8	86	PAc	9 (5 + 4)	ADT + ChT	Bicalutamide, Goserelin, Abiraterone	NEC	Prostate	36	140

^1^ HPE = Histopathological Examination| ^2^ Time elapsed until NED (in months)| ^3^ PSA = Serum Prostate Specific Antigen at NEDPC diagnosis| ^4^ PAc = Prostate Adenocarcinoma| ^5^ PMAc = Prostate Mucinous Adenocarcinoma| ^6^ Sx = Surgical/ Surgery| ^7^ RP = Radical Prostatectomy| ^8^ TURP = Transurethral Resection of the Prostate| ^9^ ADT = Androgen Deprivation Therapy| ^10^ ChT = Chemotherapy| ^11^ NEC= Neuroendocrine Carcinoma| ^12^, LCNEC: Large Cell Neuroendocrine Carcinoma.

## Data Availability

Not applicable.

## Supplementary Materials

The following are available online at https://www.mdpi.com/article/10.3390/diagnostics12061387/s1, Table S1: Neuroendocrine immunohistochemical markers. Figure S1: Overall Survival Curve. Figure S2: Time elapsed to neuroendocrine differentiaton.

## References

[1] National Cancer Institute—Surveillance, Epidemiology, and End Results Program (SEER) Cancer Stat Facts: Prostate Cancer. https://seer.cancer.gov/statfacts/html/prost.html.

[2] World Health Organization International Agency for Research on Cancer—GLOBOCAN 2020. https://gco.iarc.fr/.

[3] Barron D.A., Rowley D.R. (2012). The reactive stroma microenvironment and prostate cancer progression. Endocr. Relat. Cancer.

[4] You S., Knudsen B.S., Erho N., Alshalalfa M., Takhar M., Al-Deen Ashab H., Davicioni E., Karnes R.J., Klein E.A., Den R.B. (2016). Integrated Classification of Prostate Cancer Reveals a Novel Luminal Subtype wth Poor Outcome. Cancer Res..

[5] Nuhn P., De Bono J.S., Fizazi K., Freedland S.J., Grilli M., Kantoff P.W., Sonpavde G., Sternberg C.N., Yegnasubramanian S., Antonarakis E.S. (2018). Update on Systemic Prostate Cancer Therapies: Management of Metastatic Castration-resistant Prostate Cancer in the Era of Precision Oncology. Eur. Urol..

[6] Hu C.-D., Choo R., Huang J. (2015). Neuroendocrine Differentiation in Prostate Cancer: A Mechanism of Radioresistance and Treatment Failure. Front. Oncol..

[7] Dicken H., Hensley P.J., Kyprianou N. (2019). Prostate tumor neuroendocrine differentiation via EMT: The road less traveled. Asian J. Urol..

[8] Patel G.K., Chugh N., Tripathi M. (2019). Neuroendocrine Differentiation of Prostate Cancer—An Intriguing Example of Tumor Evolution at Play. Cancers.

[9] Beltran H., Rickman D.S., Park K., Chae S.S., Sboner A., MacDonald T.Y., Wang Y., Sheikh K.L., Terry S., Tagawa S.T. (2011). Molecular Characterization of Neuroendocrine Prostate Cancer and Identification of New Drug Targets. Cancer Discov..

[10] Papandreou C.N., Daliani D.D., Thall P.F., Tu S.-M., Wang X., Reyes A., Troncoso P., Logothetis C.J. (2002). Results of a Phase II Study with Doxorubicin, Etoposide, and Cisplatin in Patients with Fully Characterized Small-Cell Carcinoma of the Prostate. J. Clin. Oncol..

[11] Maurer T., Eiber M., Schwaiger M.E.M., Gschwend T.M.J.E. (2016). Current use of PSMA–PET in prostate cancer management. Nat. Rev. Urol..

[12] Hofman M.S., Lawrentschuk N., Francis R., Tang C., Vela I., Thomas P., Rutherford N., Martin J.M., Frydenberg M., Shakher R. (2020). Prostate-specific membrane antigen PET-CT in patients with high-risk prostate cancer before curative-intent surgery or radiotherapy (proPSMA): A prospective, randomised, multicentre study. Lancet.

[13] Gofrit O.N., Frank S., Meirovitz A., Nechushtan H., Orevi M. (2017). PET/CT With 68Ga-DOTA-TATE for Diagnosis of Neuroendocrine: Differentiation in Patients with Castrate-Resistant Prostate Cancer. Clin. Nucl. Med..

[14] Liu Y., Zuckier L.S., Ghesani N.V. (2010). Dominant uptake of fatty acid over glucose by prostate cells: A potential new diagnostic and therapeutic approach. Anticancer Res..

[15] Reinicke K., Sotomayor P., Cisterna P., Delgado C., Nualart F., Godoy A. (2012). Cellular distribution of Glut-1 and Glut-5 in benign and malignant human prostate tissue. J. Cell. Biochem..

[16] Spratt D.E., Gavane S., Tarlinton L., Fareedy S.B., Doran M.G., Zelefsky M.J., Osborne J.R. (2014). Utility of FDG-PET in clinical neuroendocrine prostate cancer. Prostate.

[17] Conteduca V., Oromendia C., Eng K.W., Bareja R., Sigouros M., Molina A., Faltas B.M., Sboner A., Mosquera J.M., Elemento O. (2019). Clinical features of neuroendocrine prostate cancer. Eur. J. Cancer.

[18] Mohler J.L., Antonarakis E.S., Armstrong A.J., D’Amico A.V., Davis B.J., Dorff T., Eastham J.A., Enke C.A., Farrington T.A., Higano C.S. (2021). Prostate cancer, version 2.2022, NCCN clinical Practice guidelines in Oncology. J. Natl. Compr. Cancer Netw..

[19] Spetsieris N., Boukovala M., Patsakis G., Alafis I., Efstathiou E. (2020). Neuroendocrine and Aggressive-Variant Prostate Cancer. Cancers.

[20] Hope T.A., Goodman J.Z., Allen I.E., Calais J., Fendler W.P., Carroll P.R. (2018). Metaanalysis of 68Ga-PSMA-11 PET Accuracy for the Detection of Prostate Cancer Validated by Histopathology. J. Nucl. Med..

[21] Sartor O., de Bono J., Chi K.N., Fizazi K., Herrmann K., Rahbar K., Tagawa S.T., Nordquist L.T., Vaishampayan N., El-Haddad G. (2021). Lutetium-177–PSMA-617 for Metastatic Castration-Resistant Prostate Cancer. N. Engl. J. Med..

[22] Taher A., Jensen C.T., Yedururi S., Surasi D.S., Faria S.C., Bathala T.K., Mujtaba B., Bhosale P., Wagner-Bartak N., Morani A.C. (2021). Imaging of Neuroendocrine Prostatic Carcinoma. Cancers.

[23] Bakht M.K., Derecichei I., Li Y., Ferraiuolo R.-M., Dunning M.J., Oh S.W., Hussein A., Youn H., Stringer K.F., Jeong C.W. (2019). Neuroendocrine differentiation of prostate cancer leads to PSMA suppression. Endocr. Relat. Cancer.

[24] Aggarwal R., Huang J., Alumkal J.J., Zhang L., Feng F.Y., Thomas G., Weinstein A., Friedl V., Zhang C., Witte O.N. (2018). Clinical and Genomic Characterization of Treatment-Emergent Small-Cell Neuroendocrine Prostate Cancer: A Multi-institutional Prospective Study. J. Clin. Oncol..

[25] Shen K., Liu B., Zhou X., Ji Y., Chen L., Wang Q., Xue W. (2021). The Evolving Role of 18F-FDG PET/CT in Diagnosis and Prognosis Prediction in Progressive Prostate Cancer. Front. Oncol..

[26] Perez P.M., Hope T.A., Behr S.C., van Zante A., Small E.J., Flavell R.R. (2019). Intertumoral Heterogeneity of 18F-FDG and 68Ga-PSMA Uptake in Prostate Cancer Pulmonary Metastases. Clin. Nucl. Med..

[27] Thang S.P., Violet J., Sandhu S., Iravani A., Akhurst T., Kong G., Kumar A.R., Murphy D.G., Williams S.G., Hicks R.J. (2018). Poor Outcomes for Patients with Metastatic Castration-resistant Prostate Cancer with Low Prostate-specific Membrane Antigen (PSMA) Expression Deemed Ineligible for 177Lu-labelled PSMA Radioligand Therapy. Eur. Urol. Oncol..

[28] Parida G.K., Tripathy S., Gupta S.D., Singhal A., Kumar R., Bal C., Shamim S.A. (2018). Adenocarcinoma Prostate with Neuroendocrine Differentiation: Potential Utility of 18F-FDG PET/CT and 68Ga-DOTANOC PET/CT Over 68Ga-PSMA PET/CT. Clin. Nucl. Med..

[29] Bakht M.K., Lovnicki J.M., Tubman J., Stringer K.F., Chiaramonte J., Reynolds M.R., Derecichei I., Ferraiuolo R.-M., Fifield B.-A., Lubanska D. (2019). Differential Expression of Glucose Transporters and Hexokinases in Prostate Cancer with a Neuroendocrine Gene Signature: A Mechanistic Perspective for 18F-FDG Imaging of PSMA-Suppressed Tumors. J. Nucl. Med..

[30] Graham M.M., Gu X., Ginader T., Breheny P., Sunderland J. (2017). 68Ga-DOTATOC Imaging of Neuroendocrine Tumors: A Systematic Review and Metaanalysis. J. Nucl. Med..

[31] Usmani S., Ahmed N., Marafi F., Rasheed R., Amanguno H.G., al Kandari F. (2017). Molecular Imaging in Neuroendocrine Differentiation of Prostate Cancer: 68Ga-PSMA versus 68Ga-DOTA NOC PET-CT. Clin. Nucl. Med..

[32] Bilen M.A., Akintayo A.A., Abiodun-Ojo O.A., Kucuk O., Carthon B.C., Schuster D.M., Parent E.E. (2020). The role of 68Ga-DOTATATE for evaluation of patients with metastatic castration-resistant prostate cancer (mCRPC). J. Clin. Oncol..

[33] Iravani A., Mitchell C., Akhurst T., Sandhu S., Hofman M.S., Hicks R.J. (2021). Molecular Imaging of Neuroendocrine Differentiation of Prostate Cancer: A Case Series. Clin. Genitourin. Cancer.

