# Supplementary material for: Multitarget Molecular Imaging in Metastatic Castration Resistant Adenocarcinoma Prostate Cancer with Therapy Induced Neuroendocrine Differentiation
Source: Diagnostics (Basel). 2022 Jun 3;12(6):1387. doi: 10.3390/diagnostics12061387 (PMC9221809; doi:10.3390/diagnostics12061387)
Supplement: Supplementary file 1 [file diagnostics-12-01387-s001.zip › diagnostics-1662198-supplementary.pdf]

Table S1 Supplementary. Neuroendocrine immunohistochemical markers.

IMMUNOHISTOCHEMICAL MARKERS			N (%)
**Performed in all patients**			
Synaptophysin (+)			8 (100%)
Chromogranin (+)			8 (100%)
**Not performed in all patients**			
**Other analysed markers**	**6 (75%)**	**Patients with no additional markers**	**2 (25%)**
PSA (–)	3 (50%)		
PSA (–) & Somatostatin (+)	1 (16.67%)		
CD56 (+), CKAE3 (+)	1 (16.67%)		
NKX3 (–) in NEC	1 (16.67%)		

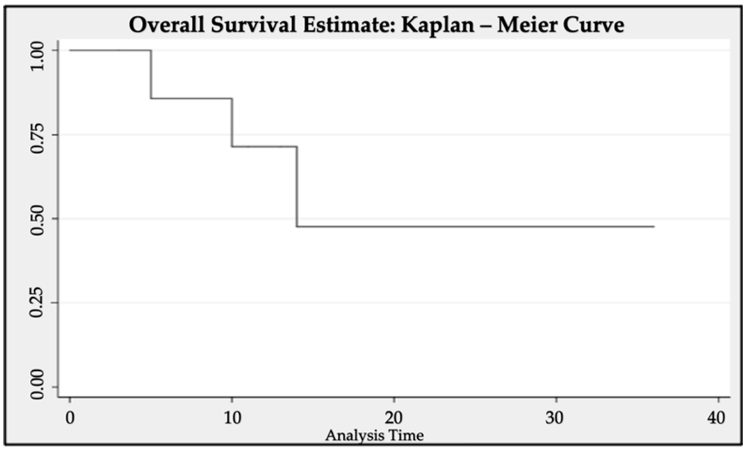

Figure S1 Supplmenetary. Overal suvival Curves after confirmation of Neurendocrine differentiation

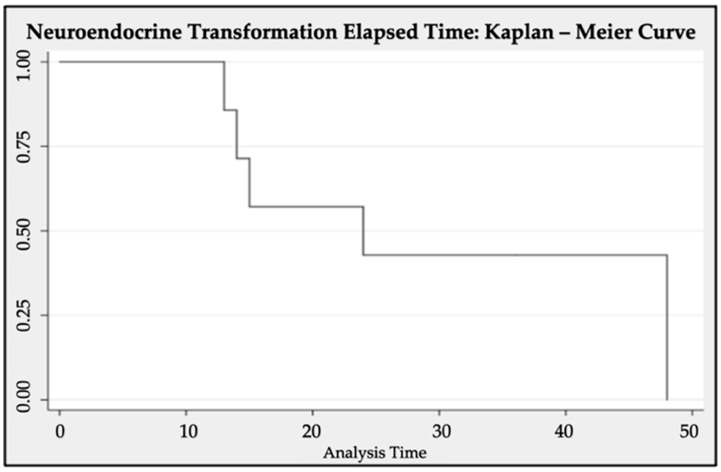

Figure S2 Supplementary. Time elapsed since diagnostic of prostate adenocarcinoma to Neurendocrine differentiation
